# Cell-Free Expression to Probe Co-Translational Insertion of an Alpha Helical Membrane Protein

**DOI:** 10.3389/fmolb.2022.795212

**Published:** 2022-02-02

**Authors:** Laura R. Blackholly, Nicola J. Harris, Heather E. Findlay, Paula J. Booth

**Affiliations:** Department of Chemistry, King’s College London, London, United Kingdom

**Keywords:** protein folding, membrane proteins, co-translational folding, cell-free, cell-free (CF) protein synthesis, lipids, membranes

## Abstract

The majority of alpha helical membrane proteins fold co-translationally during their synthesis on the ribosome. In contrast, most mechanistic folding studies address refolding of full-length proteins from artificially induced denatured states that are far removed from the natural co-translational process. Cell-free translation of membrane proteins is emerging as a useful tool to address folding during translation by a ribosome. We summarise the benefits of this approach and show how it can be successfully extended to a membrane protein with a complex topology. The bacterial leucine transporter, LeuT can be synthesised and inserted into lipid membranes using a variety of *in vitro* transcription translation systems. Unlike major facilitator superfamily transporters, where changes in lipids can optimise the amount of correctly inserted protein, LeuT insertion yields are much less dependent on the lipid composition. The presence of a bacterial translocon either in native membrane extracts or in reconstituted membranes also has little influence on the yield of LeuT incorporated into the lipid membrane, except at high reconstitution concentrations. LeuT is considered a paradigm for neurotransmitter transporters and possesses a knotted structure that is characteristic of this transporter family. This work provides a method in which to probe the formation of a protein as the polypeptide chain is being synthesised on a ribosome and inserting into lipids. We show that in comparison with the simpler major facilitator transporter structures, LeuT inserts less efficiently into membranes when synthesised cell-free, suggesting that more of the protein aggregates, likely as a result of the challenging formation of the knotted topology in the membrane.

## Introduction

Membrane proteins constitute approximately 30% of the proteome (Doerr, 2009) and command considerable attention due to their physiologically important roles and dominance of drug targets (Fagerberg et al., 2010; Lunn, 2010). Currently, most studies aimed at garnering high resolution structural or functional information on these proteins require significant amounts of pure protein sample. Classical overexpression of membrane proteins *in vivo* can result in experimental difficulties due to the complex topological nature, tedious preparation, low protein yields and potential toxicity (Wagner et al., 2007; Gubellini et al., 2011). Protein overexpressed *in vivo* can be probed using *in vitro* techniques. The details applicable to the native conformational states, folding pathways, mechanisms and functions that classical refolding *in vitro* techniques provide can be limited (Booth, 2003). In classical *in vitro* folding, a full length polypeptide chain is usually available via artificial denaturation for refolding, which is not representative of co-translational protein folding *in vivo* (Booth et al., 2001). *In vitro* investigations where a near-native lipid membrane environment is considered will provide experimental results more applicable to native protein states (Booth, 2005; Booth and Curnow, 2009). Membrane protein folding in non-native lipid environments must not overlook how the orientation and architectures of multispanning membrane proteins are determined during translation.

*In vivo*, most membrane protein biosynthesis starts on the ribosome where translation of the polypeptide chain and subsequent co-translational integration of nascent α-helices into a membrane environment is aided by the translocon (Serdiuk et al., 2019). This process occurs simultaneously whereby, in the majority of instances, a protein is co-translationally inserted *via* a membrane-embedded translocon apparatus into the bilayer during synthesis on the ribosome and as such a protein will fold upon biosynthesis (Skach, 2009; Liutkute et al., 2020). When using *in vitro* folding methods, these co-translational processes are hard to mimic (Harris and Booth, 2012; Hingorani and Gierasch, 2014). The hydrophobicity of membrane proteins, propensity to aggregation, and the chemically complex lipid composition of the surrounding native membrane environment makes experimental investigations into co-translational insertion of these proteins complicated (Bowie, 2005; Marinko et al., 2019). Probing co-translational folding is complex and the tool kits we have available to investigate this are limited.

Cell-free systems have recently provided excellent alternative techniques with the capacity to overcome the traditional problems of membrane protein production as they are not hindered by the same complications and variabilities as overexpression (Carlson et al., 2012; Findlay and Booth, 2013; Harris, 2017; Harris and Charalambous, 2018; Harris et al., 2020). We can exploit cell-free approaches to investigate membrane proteins - notably to probe co-translational folding *in vitro* as the nascent chain is being synthesized by the ribosome. These methods enable us to move from the current biophysical approaches employing artificially-denatured, full-length proteins to a situation that is more representative of cellular biosynthesis. This has afforded new opportunities to remedy the deficit of membrane protein folding studies (Schneider, 2010; Silverman et al., 2020).

Cell-free systems are frequently based on cellular extracts, such as the S30 *Escherichia coli* extract. Comprising an ensemble of the *E. coli* translation machinery, in addition to other chaperones, active enzymes and the T7 RNA polymerase to facilitate transcription, translation and protein folding *in vitro* (Kwon and Jewett, 2015; Terada et al., 2016). An issue with such extracts is that they can contain a large number of components, making it difficult to probe the influence of particular constituents (Zemella et al., 2015; Komar, 2018), as well as showing high variability (Takahashi et al, 2015), especially in extracts synthesized in non-commercial settings (Dopp and Reuel, 2018; Dopp et al., 2019).

**FIGURE 1:**
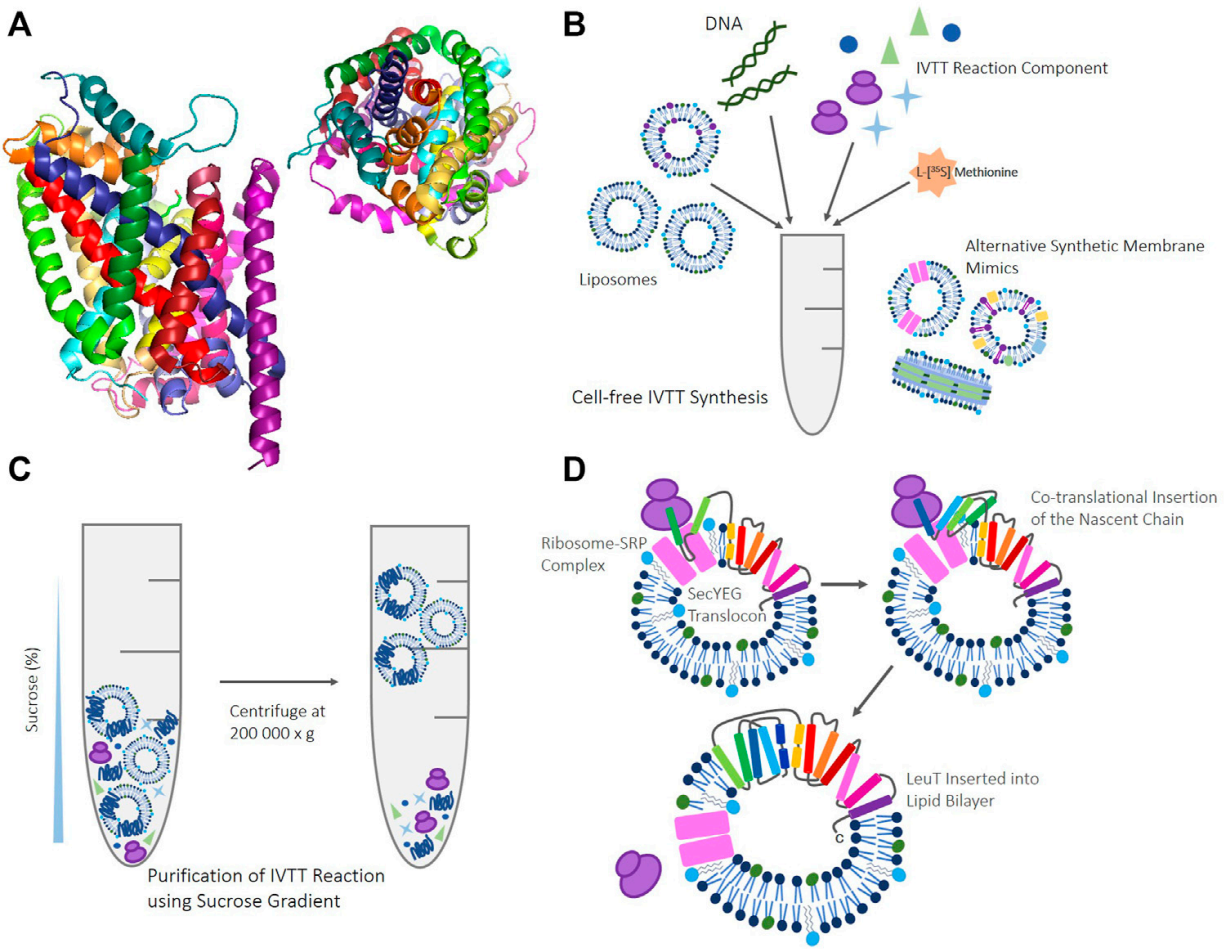
**(A)** LeuT crystal structure (3GJD) (Quick et al., 2009) in a monomeric conformation. LeuT seen from the side (left), and from above (right) to highlight the complex knotted conformation. **(B)** Schematic of reagents and components required for IVTT cell-free reactions, such as; template DNA in the form of either PCR product, RNA or dsDNA, ribosomes, rNTPs, tRNA, amino acids. In addition to IVTT components, synthetic membrane mimics like that provided by liposomes are required. Various other alternative synthetic membrane mimics can be supplemented into these reactions such as; liposomes reconstituted with other proteins, inner/inverted membrane vesicles, and nanodiscs. **(C)** Schematic of the sucrose gradient methodology (Harris, 2017) used to purify IVTT reactions. Upon completion, cell-free reactions are suspended with 60% (w/v) sucrose. 30% (w/v) sucrose and buffer are layered on top to provide gradient, before centrifugation at 200,000 x g. Proteoliposomes and empty liposomes float to the 30% sucrose: buffer interface, and any unreacted IVTT kit components and aggregated, truncated, or non-inserted protein remains at the bottom of the gradient. **(D)** Cartoon schematic to illustrate how co-translational insertion of LeuT in IVTT systems where the translocon has been reconstituted into liposomes may occur. *In vivo*, protein knotting like that seen in LeuT is thought to be established and promoted by cellular machinery, including the ribosome(Chwastyk and Cieplak, 2015), providing new folding routes (Dabrowski-Tumanski et al., 2018), modulating hydrophobic reactions (Especial et al., 2019), and stabilising folding intermediates during co-translation (Lim and Jackson, 2015b; Faísca, 2015). Translation may occur via the SecYEG translocon when reconstituted and present, but we have also shown that spontaneous insertion occurs when the translocon is absent.

Commercially synthesized extracts or cell-free systems containing purified elements tend to show less variability (Shimizu, 2001; Chong, 2014; Tuckey et al., 2014). As such, we have successfully utilized a defined system of purified components, namely the commercially available PURExpress system developed by the Ueda group (Swartz, 2001; Shimizu et al., 2005; Gregorio et al., 2019), and the Expressway kit; a commercial S30 system. PURExpress^®^ constitutes purified tRNAs, amino acids, rNTPs and other small molecules, ribosomes, the T7 RNA polymerase, aminoacyl-tRNAs, translation factors, and energy regeneration enzymes. A drawback of this particular purified system is that necessary accessory proteins like SecA, FtsY, and the signal recognition particle (SRP) are not included. In the Expressway™ commercial kit, the *E. coli* extract contains all necessary machinery required for transcription and translation, as well as accessory proteins required by the translocon. Both systems enable the addition of membrane vesicles alongside large complexes to facilitate folding, and can enable the rapid synthesis of membrane proteins, in a variety of synthetic lipid envrionments (Figure 1B) (Shimizu, 2001; Kuruma and Ueda, 2015; Khambhati, 2019).

Utilizing cell-free techniques to investigate co-translational folding and insertion of membrane proteins is an emerging field. Previous work has focused on the insertion of functional membrane proteins in liposome-assisted or nanodisc synthetic systems with cell-free methodologies, probing topogenesis using techniques such as proteolysis, substituted cysteine accessibility (SCAM), and functional assays (Sahin-Toth et al., 1995; van Geest and Lolkema, 2000; Kalmbach et al., 2007; Cappuccio et al., 2009). Further investigations are emerging, focusing on how the lipid bilayer effects nascent chain insertion using a cell-free approach, proving that these techniques are viable in probing co-translational folding *in vitro* (Roos et al., 2013; Harris, 2017; Harris and Charalambous, 2018; Sanders et al., 2018; Harris and Booth, 2019; Eaglesfield et al., 2021).

Lipid properties such as headgroup charge, mechanical properties and chain lateral pressures exerted by lipids can affect the insertion, folding and topology of a membrane protein (Bogdanov et al., 2014; Findlay and Booth, 2017; Vitrac et al., 2017). Manipulation of bilayer mimics by varying lipid composition of liposomes provided into *in vitro* transcription and translation (IVTT) cell-free systems allows for the direct investigation of lipid: protein interactions, focusing on how changes to bilayer lateral chain pressure, fluidity, polarity, charge and thickness may affect protein folding and insertion (Rigaud and Lévy, 2003; Allen et al., 2004a; Junge, 2011; Pellowe and Booth, 2019). Although liposome membrane mimics cannot be directly compared to entire membrane lipid extracts or novel polymer based nanodiscs like those produced using the developing SMALP polymer and associated techniques (Dörr, 2016; Simon et al., 2018), liposomes provide us with a system that bridges the middle ground between native environment and application of use.

Utilizing IVTT cell-free systems supplemented with liposomes also offers the potential to probe how insertion efficiency is affected by the presence of the translocon machinery. Biogenesis of nearly all alpha helical membrane proteins is governed by insertases and translocases, which provide a lower free-energy barrier for correct protein insertion and folding within a membrane (Cymer et al., 2015; Serdiuk et al., 2019). In *E. coli* this insertion is governed by SecYEG, a multimeric complex protein conducting channel (Veenendaal et al., 2004). Transmembrane helices of polytopic membrane proteins insert sequentially into the membrane utilizing the translocon machinery (Cymer et al., 2015; Pellowe and Booth, 2019; Mercier et al., 2021), and the SRP mediates ribosome targeting by coupling the synthesis of the translating nascent chain to correct cellular localization, ensuring integral membrane proteins are bought to the SecYEG translocon for correct co-translational integration into the membrane (Skach, 2011; Akopian et al., 2013; Saraogi and Shan, 2014).

SecYEG has previously been itself synthesized in PURE IVTT systems(Matsubayashi et al., 2014a; Matsubayashi et al., 2014b; Kuruma and Ueda, 2015), and liposomes containing reconstituted SecYEG have been implemented with the cell-free synthesis of the multidrug transporter EmrE (Ohta et al., 2016). We were subsequently interested to investigate the effect of the SecYEG translocon on the folding of the NSS transporter LeuT, looking at co-translational insertion utilizing reconstituted liposomes containing purified SecYEG (Figure 1D). This helps us ascertain how the presence of the translocon, with or without the necessary accessory factors, affects the overall percentage of LeuT inserted into liposomes during IVTT synthesis.

We target the leucine transporter LeuT, not previously investigated in cell-free, extending this co-translational approach to a protein with a complex topology, and advancing these successful investigations into the thermodynamics of folding in the co-translational arena. We do this employing the PURExpress^®^ and Expressway™ IVTT systems.

LeuT_Aa_ functions as a sodium/leucine transporter in *Aquifex aeolicus* and is a member of the neurotransmitter sodium symporter (NSS or SLC6) family (Yamashita et al., 2005; Krishnamurthy et al., 2009; Navratna and Gouaux, 2019). In humans, NSS proteins are responsible for the re-uptake of neurotransmitters involved in synaptic transmission (Joseph et al., 2019; Möller et al., 2019). LeuT has been widely used as a blueprint to provide insights into the organization, mechanisms and functions of mammalian NSS transporters, as it is an orthologue of eukaryotic proteins such as the Dopamine active transporter (DAT) and the Serotonin transporter (SERT) (Torres and Amara, 2007; Cheng and Bahar, 2019). Eukaryotic NSS proteins are physiologically important in humans with NSS dysfunction being related to various chronic neurological disorders such as depression, epilepsy and Parkinson’s disease (Gotfryd et al., 2020). LeuT contains 12 transmembrane helices arranged in a coupled figure-eight (4 1) trefoil (31) slipknot (Figure 1A) (King et al., 2007; Yeates et al., 2007; Sułkowska et al., 2012), which has been postulated to be important for protein stability (Sanders et al., 2018). Knotted proteins account for 1% of known high resolution structures in the Protein Data Bank (PDB), although since membrane proteins remain underrepresented in the PDB, the extent of knotting in helical membrane proteins is unknown (Lim and Jackson, 2015a; Jarmolinska et al., 2019). Studies of knotted proteins are currently focused almost exclusively on a small number of water soluble proteins and have suggested that knots may be important for activity or increasing protein stability (Sułkowska et al., 2012; Faísca, 2015; Xu et al., 2018).

The hyperthermophile *Aquifex aeolicus* from which LeuT has been derived presents diverse lipid membrane structures (Braakman and Smith, 2014; Siliakus et al., 2017). Little is known about the lipid headgroups in this eubacterial species, although they are known to be vastly different to those found in *E. coli* membranes (Sturt et al., 2004; Jain et al., 2014; Sohlenkamp and Geiger, 2015). In addition to this, other lipids associated with eubacteria are significantly more branched (Sohlenkamp and Geiger, 2015), believed to be linked to adaptations to high temperature (Koga, 2012; Siliakus et al., 2017). Lipids, in particular sphingolipids, cholesterol and other anionic lipid species, are thought to play a significant role in regards to transporter conformational dynamics, structural stabilization, and modulating substrate interactions for eukaryotic NNS transporters (Magnani et al., 2004; Hong and Amara, 2010; Khelashvili and Weinstein, 2015; Joseph et al., 2019). An example of this is cardiolipin (CL), thought to be important in the stabilization of LeuT dimers *in vitro* (Gupta et al., 2017).

Our previous work concerning the cell-free synthesis of multidomain membrane proteins has involved developing a methodology in which to purify spontaneously inserted protein from cell-free kit components, to probe the effects of the lipid bilayer on co-translational folding (Figures 1B,C). (Harris et al., 2020; Findlay and Booth, 2013; Harris, 2017; Harris and Booth, 2019; Harris and Booth, 2017) Thus far, we have successfully applied this cell-free approach to multi-domain proteins such as the rhomboid protease, GlpG (Harris, 2017), and the major facilitator transporters, LacY and XylE (Harris and Booth, 2019). Our aim is to develop these methodologies and ascertain their broader applicability. We herein discuss our studies extending this to a protein with a more complex, knotted structure.

The lipid membrane environment impacts the insertion (Meijberg and Booth, 2002; Allen et al., 2004a; Lorch and Booth, 2004), folding (Allen et al., 2004b; Seddon, 2008; Findlay and Booth, 2017; Sanders et al., 2018), and function of membrane proteins (Bogdanov and Dowhan, 1999; Lee, 2004; Lee, 2005; Bogdanov et al., 2008). We have exploited this lipid influence to optimize the efficiency of our cell-free co-translational folding systems, and to provide a system more similar to that of native membranes. Here, we were interested to explore whether manipulation of synthetic lipid mixtures is sufficient to ensure efficient co-translational insertion and folding of a protein with a complex structure, or if additional cellular factors such as the translocon would be required by LeuT for insertion in cell-free systems (Phillips et al., 2009). Notably, through manipulation of the lipid composition, we show herein that on average ≤ 24% of all LeuT protein synthesized inserts and folds correctly in a synthetic lipid membrane in IVTT systems.

## Materials and Methods

All standard reagents were purchased from Sigma. The EXPRESSway™ Mini Cell-Free Expression system was purchased from ThermoFisher Scientific. The PURExpress^®^ *In Vitro* Protein Synthesis kit and all molecular biology reagents were purchased from New England Biolabs unless stated otherwise. Methionine, L-[35S] was purchased from PerkinElmer. Lipids were purchased from Avanti Polar lipids, and the EnzCheck™ Phosphate Assay Kit, and NuPAGE Bis-Tris Gels were purchased from ThermoFisher Scientific.

Wild type LeuT_Aa_ was modified with a C-terminal 10x Histidine tag (10x His) (WT CHis-LeuT) in a pET28a vector. The LeuT gene was adapted and codon optimized for expression in heterologous *E. coli* systems using the GENEius tuning tool (Eurofins Genomics) (Sanders et al., 2018). For use in cell-free expression systems WT CHis-LeuT was further modified to include a C-terminal V5 tag (14aa) with a GSSG linker between the coding regions and the 10x His tag. SecYEHisG (donated by Prof. Ian Collinson, University of Bristol), was in a pBAD vector for *in vivo* overexpression, with a 6x His tag on the N-terminus of the SecE subunit coding region.

### Preparation of Lipids

5 mg of 1,2-dioleoyl-sn-glycero-3-phosphoethanolamine (DOPE), 1,2-dioleoyl-snglycero-3-phosphocholine (DOPC), 1,2-dioleoyl-sn-glycero-3-phospho-(1′-rac-glycerol) (sodium salt) (DOPG), *E. coli* Polar Lipid Extract, Cardiolipin (*E. coli*) (sodium salt) (CL_*E. coli*_), 1′,3′-bis[1,2-dipalmitoyl-sn-glycero-3-phospho]-glycerol (sodium salt) (CL_16:0_), 1′,3′-bis[1,2-dioleoyl-sn-glycero-3-phospho]-glycerol (sodium salt) (CL_18:1_), 1,2-diphytanoyl-sn-glycero-3-phosphocholine (DPhPC) and 1,2-diphytanoyl-sn-glycero-3-phospho-(1′-rac-glycerol) (sodium salt) (DPhPG) lipids were dissolved in cyclohexane at 45°C. Required ratios of lipids were mixed and flash frozen in liquid N_2_ before freeze drying overnight. Lipids were stored at -20°C upon removal from the freeze dryer, and N_2_ gas was passed over lipid films to prolong storage life and for preservation.

Liposomes were prepared for IVTT using preparation as previously described (Harris, 2017), and lipid films were resuspended at 10 mg ml^−1^ in 40 mM HEPES-KOH (pH 7.6) and suspensions extruded through 200 nm or 400 nm Millipore filters using a mini-extruder with a minimum of 25 pushes. Liposomes were used immediately in IVTT.

### Cell-free Synthesis and Insertion of LeuT

The PURExpress® *In Vitro* Protein Synthesis Kit and EXPRESSway™ Mini Cell-Free Expression system were used following manufacturer’s instructions. Liposomes were supplemented instead of buffer to make up to the required reaction volume, and in each case provided a final concentration of lipids at 3 mg ml^−1^ in both cell-free kits. The total reaction volume for the EXPRESSway™ commercial kit is larger than that of PURExpress^®^; 25 µl volume for PURExpress^®^, and 50 µl for EXPRESSway™. Reactions were initiated with the addition of 50 ng μl^−1^ of plasmid DNA before incubation at 30°C for 2–4 h. DNA was added after liposomes into the kit to prevent early initiation of protein synthesis. Methionine, L-[35S], 0.04–0.1 mCi ml^−1^ was added at the start of each reaction, being supplemented before the addition of plasmid DNA. Protein synthesized was quantified following the PURExpress^®^ *In Vitro* protein Synthesis Kit manual, using the Ultima Gold MV scintillation cocktail (PerkinElmer), with a 1600 TR Tri-Carb^®^ Liquid Scintillation Counter (Packard) as previously described (Harris, 2017).

#### Insertion of LeuT

All sucrose and urea solutions used were prepared in 40 mM HEPES-KOH at pH 7.6. Following IVTT using the PURExpress® *In Vitro* Protein Synthesis Kit, the reaction mix was resuspended in 80 µl 60% sucrose, before 100 µl of 30% sucrose layered on top, followed by 50 µl of 40 mM HEPES-KOH pH 7.6 buffer to complete the gradient. Gradients were then centrifuged at 200,000 x g (70,000 RPM, Beckman TLA 100 rotor) to float liposomes and proteoliposomes to the interface between the 30% sucrose: 40 mM HEPES-KOH buffer, leaving unreacted IVTT kit components to pellet to the bottom of the gradient (Figure 1C). The layers of sucrose were then separated in two aliquots; top and bottom fractions, for use in further investigation and for visualization using SDS-PAGE gel analysis as previously described (Harris, 2017). When using the EXPRESSway™ Mini Cell-Free Expression System, 200 µl 6M Urea was added to each 50 µl reaction before liposomes were pelleted via centrifugation at 350,000 x g (90,000 RPM, Beckman TLA-100 rotor) for 45 min. Liposomes were resuspended in 100 µl 60% sucrose before continuing with gradient steps as above. For detection of proteoliposomes, sucrose gradient top and bottom fractions were directly run on a 12% Nu-PAGE Bis-TRIS SDS-PAGE gel before wet transferring to a nitrocellulose membrane. Membranes were placed in a cassette with phosphor screen to develop and were imaged using a Typhoon™ FLA 7000 Biomolecular Imager. To calculate radiolabeled counts using Methionine, L-[35S], a 3 µl sample from each sucrose gradient layer, and 2 × 2 µl sample from the reaction upon completion were taken and pipetted onto MF-Millipore™ 0.5 µm membrane filter (Merck Millipore) before quantification of protein within each sample could be calculated using LSC.

#### Calculation of Insertion Efficiencies

In each lipid condition insertion efficiencies for LeuT are calculated as a percentage of protein yielded in the top fraction of the sucrose gradient after purification (Harris, 2017). This is done using protein yields obtained via LSC counts as described above. 0% insertion is where empty liposomes reside in the top fraction, and all cell-free synthesized protein aggregated in the bottom fraction, and 100% insertion is where all protein is incorporated into proteoliposomes in the top fraction, and no cell-free synthesized protein is aggregated in the bottom fraction.

The average yield from PURExpress^®^ IVTT reactions ranges between 0.1–1 µg protein per 25 µl reaction. In EXPRESSway™ a total protein yield of approximately 1–10 µg per 50 µl reaction is synthesized. Where ≥80% of total protein is lost during sucrose gradient purification, the experimental result is disregarded. There is a negative correlation between high initial IVTT expression yields and low proteoliposome recovery after purification (Supplementary Figure S4). A threshold was set so as to reject datasets where high aggregation and low protein recovery interferes with accurate calculation of any insertion efficiencies. Liposome aggregation during expression and purification is likely to reduce the amount of protein floated on the sucrose gradient. Low proteoliposome recovery, and the poor quality of such samples meant that individual IVTT reactions where ≥80% of total protein is lost during sucrose gradient purification was not compared to IVTT reactions where larger yields of protein was recovered.

Statistical analysis was performed using GraphPad Prism 9. Brown-Forsyth and Welch ANOVA tests were conducted with raw data sets when comparing average insertion efficiencies for each group of lipids investigated (DO, DPh, and CL mixes). Where individual *p* values are presented directly comparing two lipid conditions, Welch’s *t* test was calculated using complete data sets.

### Isolation and Purification of the SecYEG Translocon

A colony from a fresh transformation of pBAD SecYEG plasmid construct in c43 competent *E. coli* cells was grown in LB containing 100 μg ml^−1^ ampicillin overnight, shaking at 37°C. This was seeded into 6 x 1L smooth flasks in LB media (100 μg ml^−1^ ampicillin) and grown at 37°C, shaking, till OD ∼ 0.6–0.8 before induction with 0.1% (w/v) L-(+)-arabinose. After induction the cells were grown until stationary, and then harvested by centrifugation at 4,900 x g (4,200 RPM, Beckman JS-4.2SM) at 4°C for 45 min. Cell pellets were resuspended and washed in 200 ml phosphate-buffered saline (PBS) at 4°C at 9,000 x g (6,000 RPM, Avanti JLA-8.1000) for 10 min. Supernatant was then discarded and the pellet resuspended in 50 ml PBS with cOmplete™ protease inhibitor tablet, after which 1 µl of benzonase was added to sample, before incubation at room temperature on a roller shaker for 10 min. The sample was homogenized, and cells lysed using a constant systems cell disruptor; 1 pass, before ultracentrifugation at 148,000 x g at (38,000 RPM, Beckman Type 70 Ti rotor) 4°C, for 45 min to pellet cellular membranes. The supernatant was discarded, and the pellet was resuspended in 35 ml solubilization buffer (20 mM TRIS-HCl (pH 8), 300 mM NaCl, 10% (w/v) glycerol, 0.1 mM phenylmethylsulfonyl fluoride (PMSF), 2% (w/v) n-Dodecyl β-D-maltoside (DDM), 1x cOmplete™ EDTA-free protease Inhibitor Tablet, and 20 mM imidazole) to solubilize, left to stir at 4°C for 2 h.

For affinity purification, a HisTrap™ HP column (Ni^2+^-chelated Sepharose prep-packed, 1 ml) was connected to an AKTA prime chromatography system (GE Healthcare) equilibrated with 0:100 Buffer B: Buffer A. Buffer A: 20 mM TRIS-HCl (pH 8), 300 mM NaCl, 10% (w/v) glycerol, 0.1 mM PMSF, (0.1% w/v) DDM, Buffer B: 20 mM TRIS-HCl (pH 8), 300 mM NaCl, 10% (w/v) glycerol, 0.1 mM PMSF, 0.1% (w/v) DDM, 500 mM Imidizole, 1 mM 2-Mercaptoethanol. Solubilized, filtered sample was loaded onto HisTrap™ HP column, and after protein binding the column was washed with 10 CV 0:100 Buffer B: Buffer A, then 25 CV 15:85 Buffer B: Buffer A. Protein was eluted with 100% Buffer B and directly injected onto a HiLoad^®^ 16/600 Superdex^®^ gel filtration column before fractions collected using peak fractionation and then pooled and concentrated where required. Pooled fraction concentration determined *via* a NanoDrop™ spectrophotometer at UV280. A sample of protein was visualized using SDS-PAGE, run on a NuPAGE Bis-Tris Protein Precast gel with MES SDS running buffer, before protein was stored at −80° (Supplementary Figure S2).

#### Reconstitution of SecYEG

1.2% n-Octyl-β-d-glucopyranoside (OG) was added to liposomes pre-extruded at 400 nm diameter, prepared as described above in 40 mM HEPES pH 7.6, before incubation using a rotator mixer for 30 min at room temperature. SecYEG was then subsequently added at a predetermined concentration before further incubation in a rotator mixer for 1 h, again at room temperature. Detergent was then removed using a Pierce™ Detergent Removal Spin Column (ThermoFisher). Reconstitution efficiency and protein concentration in the liposomes was then determined using a Markwell-Lowry assay (Markwell et al., 1978) before use in subsequent cell-free experiments.

Where SecYEG was reconstituted at a SecYEG: lipid ratio (w/w) of 1:50, this equates to a composition of approximately 1.7% of heterotrimeric SecYEG per liposome, assuming a theoretical 100% reconstitution efficiency and in 400 nm liposome vesicles. For conditions where SecYEG was reconstituted into 400 nm liposomes at 1 in 25, approximately 3.4% of the liposome surface area was heterotrimeric SecYEG monomers, and at 1 in 100 only 0.86%. In IVTT where the effect of SecYEG was investigated and in liposome conditions where SecYEG was absent, liposomes were mock reconstituted with the addition and removal of detergent, following the same methodology as the SecYEG condition. Liposomes were immediately used in ATPase assay after reconstitution and in IVTT experiments.

#### SecYEG ATPase Activity

The EnzChek™ Phosphate Assay kit (ThermoFisher) was utilized to investigate SecYEG reconstituted protein when bound with SecA. SecA was donated by Prof. Ian Collinson (Collinson Group, University of Bristol) in a pET28 plasmid. SecA was purified following methods as previously described (Gold et al., 2007). The reaction mix was set up, initially omitting the experimental substrate, ATP. Reaction mix: 10 µL 20x Reaction Buffer, 40 µl MESG substrate solution, 2 µl purine nucleoside phosphorylase (PNP), 0.05–0.03 µM SecA, 50–100 µl liposomes, X µl nuclease free water (to make up 200 µl total reaction volume). Reaction mix was preincubated for 15 min at room temperature before initiation by the addition of 1 mM ATP. Sample was inverted to mix before immediately reading the A360 in a spectrophotometer as a function of time. Reactions were run for 30 min, or until no further change in A360 was observed, where no more SecA dependent ATPase activity by SecYEG present in liposomes occurred. Only functionally active SecYEG was used in insertion IVTT cell-free experiments.

### Inverted Membrane Vesicle (IMV) Preparation

Halophilic archaeal strains of *Halobacterium salinarium*, wild type (S9) and L33 (bR knockout) were grown aerobically in 50 ml basal salt liquid media, pH 7.2 (4 M NaCl, 100 mM Mg.SO_4_.7H_2_0, 10 mM Na_3_C_6_H_5_O_7_, 25 mM KCl, 1% (w/v) Oxoid peptone) at 39°C in a shaking incubator at 150 RPM till an OD (A600) of 1.0. 10 ml of preliminary culture was subsequently seeded into a 1 L flask containing 700 ml basal salt liquid media, grown as before for another 4 days or until stationary growth was reached at an OD (A600) between 1–1.5. The crude vesicles were then harvested via centrifugation at 4,900 x g (4,200 RPM, Beckman JS-4.2SM) for 45 min. The supernatant was subsequently discarded and the pellet resuspended in buffer A (50 mM Tris-HCl (pH 7.2, 1.75 M NaCl) at v:w, buffer: pelleted cell weight of 1:1. Sample homogenized using a OneShot consistent systems cell-disruptor at 4°C, 20,000 psi, performing a total of 3 passes, keeping the crude vesicle pellet on ice at all times. Homogenized sample ultracentrifuged at 170,000 x g (40,000 RPM, Beckman Type 70 Ti rotor) for 1 h at 4°C before pellet resuspended in 5 ml of buffer A. Inverted membrane vesicles were purified on a sucrose gradient. All gradient concentrations of sucrose were made up in 2 M NaCl, 50 mM Tris-HCl (pH 7.2). 1 ml of crude vesicle membrane pellet resuspension was carefully pipetted onto a sucrose cushion comprising six steps. Sucrose density gradient composition; 1.5 ml 1 (1.5 M), 2.5 ml B (1.4 M), C (1.3 M), D (1.2 M), 2.0 ml E (1.1 M), and 1 ml F (0.9 M). Gradients centrifuged at 187,000 x g (32,000 RPM, Beckman SW 32.1 Ti rotor) for 24 h at 4°C, rotor acceleration and deceleration speed set at maximum. Bands containing IMV’s were collected and diluted into 20 ml of buffer B (50 mM Tris-HCl pH 7.2, 2 M NaCl) before centrifuging at 230,000 x g (45,000 RPM, Beckman Type 60 Ti rotor) for 2 h at 4°C. Pellets resuspended again 0.5 ml of buffer B and stored at −80°C until use.

## Results

### Cell-free Synthesis and Insertion of LeuT Into Liposomes

Cell-free IVTT of LeuT was initially performed using the PURExpress^®^ system, in the presence of liposomes comprising five lipid mixes (1–5) (Table 1). Following reaction incubation and IVTT, liposomes were floated on a gradient of sucrose and the insertion efficiency was calculated. Insertion efficiency is represented as a percentage of protein in the top fraction over the total protein synthesized, using liquid scintillation counts (LSC) from incorporated Methionine, L-[35S] during protein cell-free synthesis.

**TABLE 1:** Lipid compositions and their respective molar ratios investigated for LeuT spontaneous insertion 1–10. In each lipid condition the average mean insertion efficiency is provided, calculated as a percentage of protein in the top fraction of the sucrose gradient after purification. 0% insertion is where proteoliposomes reside solely in the top fraction, and all protein aggregated in the bottom fraction, and 100% insertion where all protein is incorporated into proteoliposomes in the top fraction, and no protein aggregated in the bottom fraction. Average total yields for each condition are presented as the total amount of protein synthesized during IVTT before purification. Protein lost between the total yield and the yield after purification ranges from 0–0.25 µg, equating to an average loss of ≤ 25%. All mean insertion efficiencies are thus calculated from the total protein yield. The total protein yields after purification are presented in Supplementary Table S1. Both the average yield and mean insertion efficiency (%) were calculated from a minimum of three repeats (*n* ≥ 3), and experimental errors for mean insertion are presented as SEM. The only condition where less than 3 repeats were conducted was in the IMV condition, where *n* = 2. All experiments were conducted in PURExpress^®^ except the IMV condition where Expressway™ was used.

	*Lipid Composition*	*Lipid Molar Ratio*	*Mean Insertion (%)*	*SEM*	*Average Yield (µg/25 µl)*
*1*	DOPC	**1**	**20**	**6.9**	**0.56**
*2*	DOPC:DOPG	**50:50**	**24**	**6**	**0.62**
*3*	DOPC:DOPE	**50:50**	**18.4**	**5.8**	**0.59**
*4*	DOPE:DOPG	**50:50**	**17.6**	**3.5**	**0.61**
*5*	DOPC:DOPE:DOPG	**24.5:50.5:25**	**16.5**	**3.6**	**0.56**
*6*	DPhPC	**1**	**17.5**	**2.1**	**0.42**
*7*	DPhPG:DPhPC	**34:66**	**12.5**	**4.0**	**0.30**
*8*	DPhPG:DPhPC	**50:50**	**16.3**	**4.1**	**0.40**
*9*	DPhPG:DPhPC	**66:34**	**21.4**	**4.8**	**0.53**
					
	IMV		**19.8**	**1.9**	**2.7**

LeuT was found to insert into liposomes with a mean insertion efficiency of 16.5–24% (Table 1). This is in a consistent range with other membrane transporters previously investigated (Long et al., 2012; Ando, 2016; Harris, 2017; Harris et al., 2020). The highest percentage of spontaneous insertion occurred in liposomes with a lipid composition of a 50:50 M ratio (DOPC:DOPG) at an insertion yield of 24 ± 6%. The lowest percentage insertion efficiencies could be seen in DOPE lipid mixes; DOPC:DOPE, DOPG:DOPE, and DOPC:DOPE:DOPG at 18.4 ± 5.8%, 17.6 ± 3.5%, and 16.5 ± 3.6% respectively. Insertion of LeuT in all mixes of DOPC, DOPG, and DOPE lipids does not appear to be heavily dictated by liposome properties, and preference to a particular bilayer is not apparent. The average insertion efficiencies for the individual DiOleoyl (DO) lipid mixes tested are unlikely to be statistically significant (*p* < 0.85) where each lipid composition is directly compared.

### Insertion of LeuT Into Liposomes Constituting Near-Native Lipids

As LeuT is natively expressed in the hyperthermophillic, chemolithotrophic eubacterium; *Aquifex aeolicus* (Deckert et al., 1998; Singh and Pal, 2015), the native environment in which this protein resides is vastly different to the DO lipid environment provided in these experiments for spontaneous insertion in IVTT conditions. Isoprenoids are a major component in archaea (and eubacterium) membranes (Lange et al., 2000; Koga and Morii, 2007). Liposomes comprising DiPhytanoyl (DPh) lipids, a synthetic lipid comprising two methyl-branched acyl chains attached to a glycerol moiety to mimic the lipid physiological properties of both eubacterial and eukaryotic origin (Panov et al., 2014; Tsuchikawa et al., 2020), were subsequently employed. These lipids have been shown to produce a stable planar lipid membrane, and DPhPC forms excellent stable model bilayers (Hung et al., 2000). Various liposome conditions comprising branched lipids were studied for LeuT insertion to mimic a nearer-native membrane environment (6–10) (Table 1).

The percentage insertion of LeuT into liposomes containing DPh lipids remains in a comparable range to that with DO lipids; between 12–22% (Table 1). There is no statistical difference in the mean insertion efficiencies for these lipid conditions (*p* < 0.80). The highest insertion efficiency into DPh containing liposomes is seen in 66:34 DPhPG:DPhPC at 21.4 ± 4.8%. Insertion into 50:50 DPhPG:DPhPC liposomes was lower than 50:50 DOPG:DOPC liposomes at 16.3 ± 4.1%. The insertion efficiency into pure DPhPC is also lower than pure DOPC liposomes, where the percentage mean insertion efficiency for DPhPC was 17.5 ± 2.1% compared to 20 ± 6.9% in DOPC albeit these results are not within error.

The total protein yields were lower than that seen with DO lipids (Table 1). This was particularly apparent with 100% DPhPG liposomes where only 0.06–0.19 µg of total protein was synthesized in each IVTT reaction, notably lower compared to 0.3–72 µg in other lipid conditions (Supplementary Table S2). Such low yields were not reliably quantifiable above background, therefore 100% DPhPG insertion efficiencies were not compared further to conditions where protein IVTT expression yields were higher.

### Insertion of LeuT Inverted Membrane Vesicles

To further investigate if a nearer-native lipid environment would increase the percentage of protein to spontaneously insert during IVTT, inverted membrane vesicles (IMVs) from *Halobacterium salinarium* L33 were produced. Although *H. salinarium* is a halophilic archaeon and not considered hyperthermophillic nor chemolithotrophic like *A. aeolicus*, this extremophile can be cultivated and grown using relatively standard, well documented lab conditions (Ring and Eichler, 2001; Eichler, 2019; Vauclare et al., 2020). The lipid environment of IMVs was hoped to provide a nearer-native environment than that provided by DO and DPh lipid compositions.

Purified *H. salinarium* IMVs were supplemented into the Expressway™ system, where the sucrose gradient was adapted for variability in IMV flotation as noted in methods. Spontaneous insertion of LeuT into IMVs using LSC counts gave a percentage insertion of 19.8 ± 1.9% (Table 1), which initially appears consistent with other IVTT reactions in Expressway™ (Table 2). It should be noted that the 2.7 µg yield of average total protein calculated using LSC counts cannot be distinguished as corresponding solely to spontaneously inserted LeuT, on account of non-specific reactions with Methionine, L-[35S] and IMV components (Supplementary Figure S1). Optimizing the use of IMVs in IVTT was thus not undertaken and different avenues of investigation were pursued.

**TABLE 2:** Lipid compositions and their respective molar ratios investigated for LeuT spontaneous insertion in PURExpress^®^ 1–6. Mean insertion efficiencies (%) and average total yields with each condition are shown for each lipid condition tested. The total protein yields after purification are presented in Supplementary Table S3. The average protein yield after sucrose gradient purification is consistent with the total yields immediately after IVTT reaction, so protein is not lost during purification steps. The average total yield and mean insertion efficiencies (%) were calculated from three repeats (*n* = 3) except condition 6 where *n* = 5. Experimental errors for mean insertion are presented as SEM.

	*Lipid Composition*	*Lipid Molar Ratio*	*Mean Insertion Efficiency (%)*	*SEM*	*Average Yield (µg/25 µL)*
*1*	**DOPC:CL**_***E. coli***_	99.5:0.5	**8.3**	**1.0**	**0.55**
*2*	**DOPC:CL**_***E. coli***_	95:5	**12.9**	**1.8**	**0.56**
*3*	**DOPC:DOPE:DOPG:CL**_***E. coli***_	24:51.5:24:0.5	**13.9**	**1.6**	**0.57**
*4*	**DOPC:DOPG:CL**_**18:1**_	50.5:49:0.5	**19.1**	**1.7**	**0.60**
*5*	**DOPC:DOPG: CL**_**18:1**_	53:41.5:5.5	**16.2**	**1.9**	**0.62**
*6*	**DOPE:DOPG:CL**_**18:1**_	72:27.5:0.5	**17.4**	**3.7**	**0.72**

### Insertion of LeuT Into Cardiolipin Containing Liposomes

The effect of CL on the spontaneous insertion of LeuT in IVTT was also investigated as CL is postulated to play a stabilizing role in LeuT dimers (Gupta et al., 2017; Corey et al., 2019). CL has also been implicated in playing a stabilizing role for other membrane proteins, including the Na^+^/H^+^ antiporter NhaA (Gupta et al., 2017; Rimon et al., 2019). Two commercially available CL extracts were used to investigate this effect: natural *E. coli* CL (CL_*E. coli*_) and purified trans18:1 CL (CL_18:1_). The former has a varied fatty acid distribution within the natural extract, although the two most common acyl chain structures present are 16:0 (33.3%) and cyclo17:0 (27%), with trans18:1 representing 14.4% of this natural lipid extract mixture (Macias et al., 2019). DO lipid mixes at ratios previously investigated were doped with 0.5–5% of either CL_*E. coli*_ or CL_18:1_ to investigate any effects on spontaneous protein insertion. Liposomes constituting various lipid mixes were supplemented into IVTT (Table 2). 72:22.5:5.5 DOPE:DOPC:CL_18:1_ was a particularly interesting composition to test as is comparable to that of an *E. coli* total lipid extract (Raetz and Dowhan, 1990; Sohlenkamp and Geiger, 2015; Hoyo et al., 2020).

**FIGURE 2:**
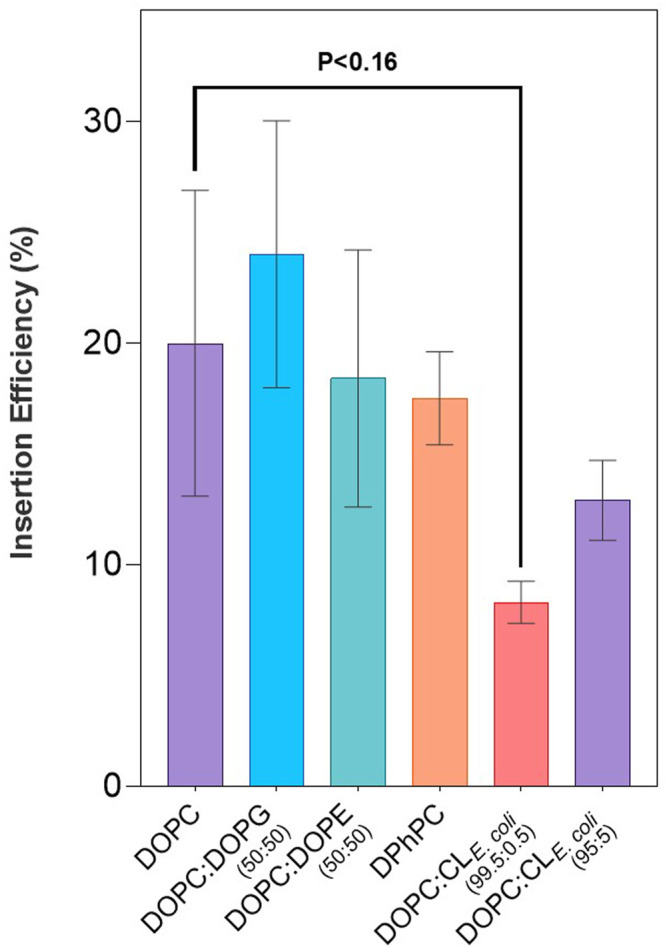
LeuT expressed in PURExpress^®^ IVTT systems in the presence of liposomes comprising various lipid compositions as presented in Table 1 and Table 2. Spontaneously inserted protein was quantified using LSC counting of Methionine, L-[^35^S] incorporated into LeuT. Insertion efficiency is represented as a percentage of protein in the top fraction over the total yield of protein synthesized. Highlighted here are the results of lipid optimization on LeuT spontaneous insertion efficiencies, comparing various lipid species derived from DO, DPh and Cardiolipin liposome compositions. Errors presented are SEM calculated from ≥3 repeats.

Spontaneous insertion of LeuT into CL liposome mixes remained consistent, albeit slightly lower than that seen with DO lipids, and with greater variability comparatively across individual CL lipid conditions (*p* < 0.09) (Figure 2). Percentage insertion of LeuT into CL lipid mixes was found to be between 8–19% (Table 2). This general decrease in insertion can be observed in direct comparison with DO lipid mixes with and without doped CL. The most significant effect can be seen with pure DOPC bilayers where spontaneous insertion is reduced 8.3 ± 1% with 0.5% CL_*E. coli*_ (*p* < 0.16) (Figure 2). The reduction in spontaneous insertion from lipid mixes without CL to with CL is less prominent with 5% CL_*E. coli*_ where spontaneous insertion is reduced from 20 ± 6.9% in pure DOPC bilayers, to 12.9 ± 1.8%, and in 50:50 DOPC:DOPG bilayers, where the percentage insertion is 24 ± 6%, compared to 19.1 ± 1.7% in 50.5:49:0.5 DOPC:DOPG:CL_18:1_ and 16.2 ± 1.9% in 53:41:5.5 DOPC:DOPG:CL_18:1_ (*p* < 0.27–0.47). CL with a saturated acyl chain structure of CL_18:1_ does not appear to reduce the spontaneous insertion of LeuT as much as that seen with natural CL_*E. coli*_, containing a mixed distribution of acyl chain structures.

### SecYEG Reconstituted Liposomes to Improve LeuT Insertion in Cell-free Systems

The EXPRESSway™ system was used for investigations into the effect of the SecYEG translocon on LeuT insertion over the PURExpress^®^ system employed for earlier lipid optimization investigations. This system was chosen to investigate the effect of the translocon on LeuT insertion as it is an *E. coli* extract, and as such, contains all the necessary cellular machinery required for transcription and translation, as well the associated accessory proteins required by the translocon for co-translational insertion.

SecYEG was prepared (Collinson et al., 2001) and reconstituted into liposomes at various concentrations. Liposomes contained differing amounts of CL to test the efficiency of insertion in the presence of SecYEG (Table 3). CL is required for the *in vivo* stability and function of the bacterial translocon, as well as DOPG (Collinson, 2019; Ryabichko et al., 2020). SecYEG activity in reconstituted liposomes was investigated using an ATPase assay, yielding consistent activity comparable with other investigations (Supplementary Figure S3, Supplementary Table S8) (Robson et al., 2009). Lipid mixes containing SecYEG were supplemented into IVTT reactions.

**TABLE 3:** The individual lipid compositions used for conditions where SecYEG was either present or absent 1–4. SecYEG was reconstituted at a ratio of 1 in 50 (w/w) protein: lipid in conditions where present. Average total yields refer to the total amount of protein synthesized during IVTT, and average yield purified refers to the amount of protein recovered after sucrose gradient purification. We can consider the protein lost between synthesis and purification to be aggregated or non-inserted. The mean insertion efficiencies (%) are presented for each condition with SecYEG absent or present. Errors presented are SEM calculated from a minimum of 3 repeats, any experimental results disregarded can be found in Supplementary Table S5 and S6. All experiments were conducted in Expressway™.

	*Lipid Composition*	*Lipid Molar Ratio*	*SecYEG*	*Mean Insertion Efficiency (%)*	*SEM*	*Average Total Yield (µg/50 µL)*	*Average Yield Purified (µg/50 µL)*
*1*	**DOPE:DOPG:CL**_**18:1**_	72:27.5:0.5	**no**	**18.7**	**2.7**	**4.6**	**1.4**
*1*	**DOPE:DOPG:CL**_**18:1**_	72:27.5:0.5	**yes**	**20.1**	**1.1**	**4.4**	**1.4**
*2*	**DOPE:DOPG:CL**_**18:1**_	72:22.5:5.5	**no**	**23.8**	**2.9**	**4.3**	**1.7**
*2*	**DOPE:DOPG:CL**_**18:1**_	72:22.5:5.5	**yes**	**21.8**	**2.4**	**4.4**	**1.4**
*3*	**DOPE:DOPG:CL**_***E. coli***_	72:27.5:0.5	**no**	**20.3**	**2.1**	**4.2**	**1.9**
*3*	**DOPE:DOPG:CL**_***E. coli***_	72:27.5:0.5	**yes**	**16.3**	**5.9**	**4.2**	**1.5**
*4*	**DOPE:DOPG:CL**_***E. coli***_	72:22.5:5.5	**no**	**28.3**	**3.7**	**4.0**	**1.7**
*4*	**DOPE:DOPG:CL**_***E. coli***_	72:22.5:5.5	**yes**	**21.1**	**0.1**	**2.3**	**0.8**

The percentage insertion of LeuT in EXPRESSway™ remains consistent with efficiencies seen in PURExpress^®^ IVTT systems, where the insertion was between 16–28% in both “empty” and SecYEG conditions (Table 3). In all lipid conditions, insertion was not improved by the presence of SecYEG, where SecYEG was reconstituted at a 1 in 50 (w/w) protein: lipid ratio. For each condition investigated, the presence of the SecYEG translocon did not alter the insertion efficiencies, and any changes are not likely to be statistically significant (*p* < 0.14–0.64). In 72:22.5:5.5 DOPE:DOPG:CL_*E. coli*_, the insertion efficiency of 28.3 ± 3.7% was reduced to 21.1 ± 0.1% in SecYEG containing liposomes (Table 3).

### Increased SecYEG Reconstitutions to Improve LeuT Insertion in Cell-free Systems

SecYEG did not induce a significant improvement of LeuT insertion into liposomes in IVTT systems. To further investigate the effects of SecYEG on spontaneous insertion, SecYEG was reconstituted into liposomes at both 1 in 25 and 1 in 100 (w/w) protein: lipid ratios.

**FIGURE 3:**
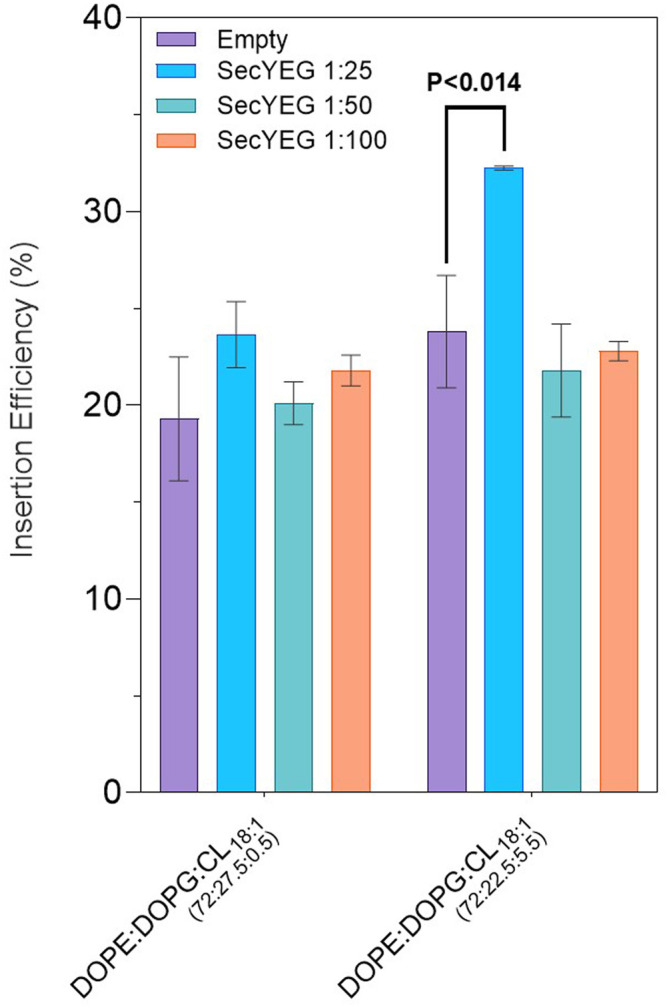
LeuT expression in EXPRESSway™ IVTT systems with SecYEG reconstituted liposomes, as presented in Table 3 and Table 4. CL_18:1_ was added into DOPE:DOPC lipid mixes at either 0.5 or 5.5% concentrations. In empty conditions, SecYEG was absent. In SecYEG conditions, SecYEG was reconstituted at a 1 in 25, 1 in 50, or 1 in 100 (w/w) protein: lipid concentration. Errors presented are SEM calculated from ≥2 repeats for 1 in 25 and 1 in 100 SecYEG conditions, and from ≥5 repeats for all other conditions.

In both lipid mixes where SecYEG was reconstituted at 1 in 25 (w/w) protein: lipid ratio, there was an increase in LeuT insertion (Table 4). In 72:27.5:0.5 mol ratios of DOPE:DOPG:CL_18:1*,*_ we see a spontaneous insertion of 23.6 ± 1.7% for 1 in 25 SecYEG conditions, compared to 20.1 ± 1.1% and 21.8 ± 0.8% for 1 in 50 and 1 in 100 conditions respectively (*p* < 0.17–0.31). In 72:22.5:5.5 DOPE:DOPG:CL_18:1_, we see a similar, yet more pronounced effect where spontaneous insertion is found to be at 32.3 ± 0.1% for 1 in 25 SecYEG, compared to 21.8 ± 2.4% and 22.8 ± 0.5% for 1 in 50 and 1 in 100 conditions respectively (Table 4). The increase in insertion for 1 in 25 SecYEG in this lipid condition is statistically significant (*p* < 0.040) when compared with empty liposomes of the same composition (Figure 3). In addition to the mean insertion rates, the yield of protein produced remains consistent across all SecYEG reconstitutions and are comparable to other EXPRESSway™ IVTT yields.

**TABLE 4:** Investigations were conducted in lipid conditions comprising 72:27.5:0.5 and 72:22.5:5.5 mol ratios of DOPE:DOPG:CL_18:1_, with each protein: lipid ratio of SecYEG. The mean insertion efficiencies (%) are presented for each lipid condition where SecYEG is present at each concentration. Errors presented are SEM calculated from a minimum of 2 repeats. The total protein yields after purification are presented in Supplementary Table S4. All experiments were conducted in Expressway™.

	*Lipid Composition*	*Lipid Molar Ratio*	*SecYEG*	*Mean Insertion Efficiency (%)*	*SEM*	*Average Total Yield (µg/50 µL)*
1	**DOPE:DOPG:CL**_**18:1**_	72:27.5:0.5	**1 in 25**	**23.6**	**1.7**	**3.8**
1	**DOPE:DOPG:CL**_**18:1**_	72:27.5:0.5	**1 in 50**	**20.1**	**1.1**	**4.4**
1	**DOPE:DOPG:CL**_**18:1**_	72:27.5:0.5	**1 in 100**	**21.8**	**0.8**	**4.4**
2	**DOPE:DOPG:CL**_**18:1**_	72:22.5:5.5	**1 in 25**	**32.3**	**0.1**	**4.0**
2	**DOPE:DOPG:CL**_**18:1**_	72:22.5:5.5	**1 in 50**	**21.8**	**2.4**	**4.4**
2	**DOPE:DOPG:CL**_**18:1**_	72:22.5:5.5	**1 in 100**	**22.8**	**0.5**	**4.7**

## Discussion

The lipid compositions chosen for study struck a balance between lipids that would yield the formation of a stable bilayer, lipids required for SecYEG functionality, and lipids that would provide a membrane mimic suitable for protein insertion (Van Dalen and De Kruijff, 2004; Phillips et al., 2009; Sachse et al., 2014; Kuruma and Ueda, 2015). Insertion of LeuT was independent of the lipid compositions investigated, where changes in lateral pressure and charge had little to no effect. We show that percentage insertion remains consistent in DO lipid mixes, where insertion appeared to not favor any of the compositions tested. This lack of dependence on membrane lipids remains in the DPh lipids tested, where changes in bilayer properties with the addition of DPhPG to DPhPC made little difference to LeuT insertion. In contrast to this we show that liposomes containing small amounts of CL such as 0.5 and 5.5%, appeared to be less favored for LeuT insertion, especially when directly compared to DO lipid mixes. Improvement of LeuT insertion was not obtained; regardless of lipid composition, or *via* the presence of the bacterial translocon SecYEG at low concentrations.

The lack of lipid dependency contrasts with previous *in vitro* folding work with LeuT that was partly denatured in the presence of urea, where unfolding was measured in reconstituted proteoliposomes. Both lateral chain pressure and the presence of negatively charged headgroups was found to increase the thermodynamic stability of LeuT and the unfolding free energy in liposomes (Sanders et al., 2018). The results we show for LeuT also contrasts with other membrane proteins investigated using this IVTT methodology which exhibit greater dependence on the lipid membrane environment provided (Findlay and Booth, 2013; Harris, 2017; Harris et al., 2020). The percentage incorporation of LeuT into proteoliposomes was lower than other transporters such as XylE and LacY, where ≥ 5% improvement in insertion can be obtained through lipid optimization. It was shown that spontaneous insertion of LacY increased from 17.0 ± 2.7% in pure DMPC bilayers, to 25.9 ± 1.6% in pure DOPC bilayers, and then to 32.7 ± 1.7% in 50:50 DOPC:DOPE bilayers, suggesting that an increase in lateral pressure led to an improvement in the insertion yield. Conversely with XylE, spontaneous insertion was lowest in pure DOPC liposomes. Insertion efficiencies were reduced as lateral pressure was introduced by the addition of DOPC, and the greatest insertion efficiency was in pure DOPG bilayers where a spontaneous insertion of 58.7 ± 2.4% was seen (Harris et al., 2020).

There is a comparative difference between higher insertion yields previously seen with major facilitator superfamily (MFS) transporters, and lower yields seen with LeuT. This may be due to an increased propensity for aggregation during insertion linked to the complexity in LeuT conformation and the knotted topology (Harris, 2017; Harris and Booth, 2019). The lack of improvement of insertion efficiencies through lipid optimization may be due to the complex structure of LeuT hindering insertion. In addition to this, LeuT is derived from eubacterium whereas the other transporters previously investigated are integral membrane proteins found in *E. coli*. The native membrane conditions required by such proteins are thus inherently different and we are limited further by the complexity in simulating a near-native environment for LeuT. More is known about the lipid architectures of *E. coli*, and much less about the lipid species and arrangements in the eubacterium *A. aeolicus*.

Further investigations into function would be required to investigate the functionality of cell-free synthesized and spontaneously incorporated protein. The low yields obtained for LeuT when synthesized in cell-free IVTT systems mean that functional assays are not viable methods to probe folding and activity. With yields of ≤ 0.7 μg (PURExpress^®^) or ≤ 2.3 µg (EXPRESSway™) per reaction of purified LeuT proteoliposomes, it was not feasible to calculate protein activity using the functional assay as previously devised for LeuT where at least 40 μg of protein was reconstituted into liposomes for each assay (Sanders et al., 2018). Despite this, previous work with other membrane proteins such as GlpG, DsbB and LacY has shown that functional protein can be synthesized in cell-free systems using our methodologies (Findlay et al., 2016; Harris, 2017). Radiolabeled gels or western blot analysis can also be used to qualitatively ascertain the quality, purity and overall proportion of spontaneously inserted protein. Our previous work has shown that protein can be considered to be folded, and therefore likely to be functional, when incorporated into proteoliposomes, present in the top fraction on a gel after sucrose gradient purification (Harris, 2017).

Low yields of protein synthesised in IVTT are incompatible with our method of quantifying insertion efficiencies using LSC counts, as these cannot distinguish above background (Supplementary Table S2). In Expressway™, low amounts of inserted protein were recovered in sucrose gradient fractions with cardiolipin containing liposomes. In some cases, as much as ∼95% of the protein is lost. The quality of some CL samples with very low protein recovery meant that the data could not be compared, and therefore for our analysis we used a cut-off of (80%) recovered protein. This is highlighted by the negative correlation seen with high initial total protein yields, and low protein yields recovered after gradient purification in EXPRESSway™ (Supplementary Figure S4), with liposomes containing cardiolipin, in particular CL_*E. coli*_ lipid species. This effect is not observed with cardiolipin containing liposomes in the PURExpress^®^ system so is a result of an interaction between the liposomes and the reaction components (Supplementary Figure S5).

In EXPRESSway™, protein yields tend to be higher, possibly as a result of larger reaction volumes. However, because the yield of protein recovered in the gradient after purification remains consistent across both IVTT systems, it shows that high initial yields do not always equate to more inserted protein being produced. IVTT systems still prove to be useful methodologies to probe protein insertion into liposomes in a range of lipid compositions (Cheng and Bahar, 2019; Chong, 2014; Collinson, 2019; Harris and Booth, 2017; Quick et al., 2009).

### LeuT Lipid Dependency

Liposomes comprising DO lipids enabled us to probe the various effects lipids can have on the synthetic lipid bilayer, and to measure what effect this has on LeuT insertion efficiency. For example, DOPC lipids assemble naturally into bilayers, and DOPE lipids do not, instead forming non-lamellar structures. Using DOPC as a standard for comparison, this lipid species has a neutral headgroup, unsaturated fatty acid chains and forms fluid bilayers. Introducing DOPE into a bilayer of DOPC is known to increase the outward lateral chain pressure, and to reduce the laterally exerted pressure in the headgroup region to create bilayers that show an increase in activation energy associated with insertion of a protein helix across a bilayer (Lorch and Booth, 2004). Addition of DOPG, a lipid species with a negatively charged headgroup introduces charge to the bilayer without significantly impacting bilayer fluidity or lateral chain pressure. Such manipulations of bilayer properties were hypothesized to affect the spontaneous insertion of LeuT, and yet we find little dependence of insertion yield on lipid composition when either DOPG or DOPE are introduced into the bilayer (Figure 2).

DPh lipids were used to provide bilayers with nearer-native properties to the *A. aeolicus* eubacterium. In these lipid conditions, low yields and an absence of improvement for insertion efficiency was seen. Although DPh branched lipids have a shorter fatty acid chain tail length, the thickness of bilayer produced is comparable to that of DO lipid bilayers (Tristram-Nagle et al., 2010; Kučerka et al., 2011). DOPC and DPhPC bilayers yielded comparable percentage insertion of LeuT (Figure 2). In DPhPG, the average initial total protein yield was significantly lower than yields seen in other DPh lipid mixes suggesting inhibition of protein expression when using pure DPhPG. A lack of lipid dependency is further illustrated by LeuT insertion into IMVs, where no improvement was present. The 2.7 µg of total protein in this condition (Table 1) is comparatively low to other total protein yields in Expressway™ (Table 3), even when we consider that these LSC counts correspond to both spontaneously inserted LeuT as well as other IMV components.

In PURExpress^®^, addition of cardiolipin species hindered LeuT insertion (Table 2). Comparing pure DOPC lipid bilayers to liposomes comprised of DOPC and either 0.5% or 5.5% CL_*E. coli*_, there is a decrease in LeuT insertion efficiency (Figure 2). Anionic phospholipids, such as CL, are understood to have a higher tendency to aggregate within IVTT buffers (Kuruma and Ueda, 2015), which is likely to increase the amount of protein lost during purification. With 0.5 and 5.5% concentrations of CL, the direct propensity for aggregation on insertion efficiency is likely to be minimal in PURExpress^®^. It would be more reasonable to suggest that the result seen with certain species of CL show that CL is less favored for insertion by LeuT. We would expect to see a marked decrease in insertion in liposome conditions containing 5.5% CL when compared with 0.5% CL if this was the case. CL_*E.*__*coli*_ comprises a mix of various CL species, containing only 14% CL_18:1_. If insertion is less negatively affected when this lipid is present than when other CL species in the natural CL extract are present, this may account for the increase in insertion efficiency between 0.5 and 5.5% CL_*E.*__*coli*_, as more CL_18:1_ would be present in the latter liposome composition.

In the natural CL_*E.*__*coli*_ extract the general alkyl chain length of fatty acid tails is ≤ 19 carbons, with the most prevalent CL species being 16:0 at 33% and cyclo17:0 at 27%. Purified 16:0 CL (CL_16:0_) was tested in EXPRESSway™ at 0.5 and 5.5% concentrations with and without SecYEG, but there was poor proteoliposome recovery after purification (Supplementary Table S7), as seen with other CL_*E. coli*_ IVTT reactions. These low recovery yields meant that LeuT insertion could not be reliably compared to the other samples in EXPRESSway™. An increase in sample loss during purification was a feature of all liposome reactions using this IVTT kit, however the type of CL present had some impact, with CL_18:1_ least affected. As cardiolipin is essential to the function of many membrane proteins, including the translocon (Collinson, 2019; Ryabichko et al., 2020), it is not always possible to do without it entirely. Where required, optimizing CL type to reaction conditions can help reproducibility.

### SecYEG Effects on LeuT Insertion

Our previous work utilizing IVTT for the synthesis of membrane proteins has focused on spontaneous insertion in the absence of the translocon machinery. We were interested in whether insertion could be improved above that of bilayer manipulations, through the reconstitution of SecYEG into liposomes supplemented into cell-free IVTT systems. Reconstitution of SecYEG for investigating the effect of the translocon on LeuT insertion needed to strike a balance between a high enough concentration to yield an effect, whilst also leaving enough surface lipid so as not to restrict incorporation *in vitro*.

Initial experiments with SecYEG showed no improvement in the insertion efficiency of LeuT during IVTT. The question this poses is thus; does the presence of SecYEG directly influence or inhibit LeuT incorporation into liposomes, or does the presence of SecYEG induce other effects which make the liposomes more or less suitable for spontaneous incorporation? This would equally remain a point of contention if the results showed an improvement in LeuT insertion. Although SecYEG is present in our membrane mimics, we cannot conclude that LeuT insertion is being facilitated *in vitro* and indeed using this protein conducting channel for insertion. Incorporation may still occur spontaneously, avoiding the translocon altogether. It may be that reconstituted SecYEG prompts a change in the surrounding lipids, affecting the fluidity and or curvature of the liposome lipid bilayer, which in turn may be behind any observed changes to the final percentage insertion of LeuT. Although this is less likely as LeuT shows little lipid dependency. Another important point to address would be that SecYEG is part of the *E. coli* translocon machinery, and is thus non-native to the eubacterium *A. aeolicus*, even as eubacterial proteins are transported through a homologous complex (Trueman et al., 2012).

Our results showed that higher concentrations of reconstituted SecYEG (1 in 25 w/w protein: lipid) in some lipid conditions improved insertion. We highlight that 5.5% CL_18:1_ yielded the best improvement in LeuT insertion when the SecYEG translocon was present at the highest protein: lipid ratio investigated, where we saw the highest insertion efficiency for LeuT at 32.3 ± 0.1% (Figure 3) (Table 4). All other concentrations of SecYEG in both cardiolipin species lipid mixes showed no improvement in LeuT insertion, and in CL_*E. coli*_, spontaneous insertion appeared hindered by the presence of SecYEG (Table 3). Is the effect of SecYEG on insertion concentration dependent? When a certain concentration of SecYEG is present, is LeuT more efficiently and effectively incorporated into proteoliposomes, or, does having more or less SecYEG per liposome alter conditions for bilayer insertion? Any lipid effects instigated by the presence of SecYEG could change when variable amounts of SecYEG is reconstituted per liposome, which would require further detailed study.

## Conclusion

We have shown that it is possible to use an *in vitro* cell-free approach for studies on the co-translational insertion of LeuT, which extends the applicability of this cell-free method to an important membrane protein transporter class. The resulting yields of insertion of LeuT are however lower than those previously reported for other membrane proteins. This may be due to the lipid composition being suboptimal and/or the knotted structure of LeuT. LeuT is a topologically complex protein that does not definitively require SecYEG for incorporation into liposomes under the conditions that were probed here. It is possible that the thermodynamics of insertion are as such that if a membranous environment is present, regardless of a complex topology, insertion is more favorable than aggregation in solution.

It appears that LeuT is also resilient to perturbations in the lipid composition, although further studies into a wider range of lipid compositions may start to reveal a dependence. Our results showed that co-translational insertion efficiency could not be improved above 24 ± 6% regardless of the lipid composition in the liposomes tested. The low insertion efficiencies can equally be explained by the minimal cell-free *in vitro* conditions not being optimized for protein insertion. We show that for DO and DPh lipids the insertion of LeuT remains consistent, and we see a general decrease in these efficiencies when CL is added at the concentrations we tested. In addition to this we show that the presence of the bacterial translocon did not consistently improve the insertion efficiency of LeuT, a result that was unexpected owing to the complex topological nature of this transporter. We can however increase the insertion efficiency to 32.3 ± 0.1% with the addition of SecYEG, above what can be achieved with lipid optimization. This builds upon previous work investigating the lipid dependency of other transporters insertion in IVTT cell-free, and contrasts with this former work which showed a high dependency on lipid environment (Harris et al., 2020; Harris, 2017; Harris and Booth, 2012; Harris and Booth, 2019).

Insertion was less favored when we included nearer-native lipids into our liposome membrane mimics. As so little is known about the lipid species and compositions of eubacterial membranes, our synthetic mimics are unlikely to be entirely representative to the native membrane environment of *A. aeolicus*. The membrane lipid architectures and structures favored by LeuT may be different to those tested here, or LeuT may have less dependance on an optimal lipid composition for insertion and folding. Lipids are known to play key roles in modulating protein insertion folding and function and we anticipate that while LeuT inserted is likely to be folded, this folding is likely to be fine-tuned by a lipid environment more native than that tested within the scope of this investigation.

We build upon our previous investigations using cell-free IVTT systems, expanding to include the first such study on a transporter with a topologically complex structure. By finding new avenues in which to probe this protein *in vitro*, we are expanding the toolkits available to investigate the co-translational folding of more complex proteins, adding to the knowledge of how these proteins may fold *in vivo*.

## Data Availability

The raw data supporting the conclusion of this article will be made available by the authors, without undue reservation.
