# Supplementary material for: Cell-Free Expression to Probe Co-Translational Insertion of an Alpha Helical Membrane Protein
Source: Front Mol Biosci. 2022 Feb 2;9:795212. doi: 10.3389/fmolb.2022.795212 (PMC8847741; doi:10.3389/fmolb.2022.795212)

Supplementary Material

1 Supplementary Tables

1.1 Table 1.

Average total yields and average purified yields for DO and DPh lipid mixes. Immediately after IVTT, total protein yields were calculated. This can be compared to the amount of protein present after sucrose gradient purification, presented here as the purified yield. Yields in both columns are presented as averages, taken from repeats of the same lipid composition. In both instances yields were calculated using LSC radiolabeled counts from Methionine, L-[^{35}S] incorporation into LeuT during cell-free synthesis. In the lipid conditions presented here all yields are μg/ 25 μL, as this is the reaction size of the PURExpress® kit used in these experiments.

	Lipid Composition	Molar Ratio	**Average Total Yield (μg / 25 μL)**	**Average Purified Yield (μg/ 25 μl)**
1	DOPC	1	**0.56**	**0.56**
2	DOPC:DOPG	50:50	**0.62**	**0.50**
3	DOPC:DOPE	50:50	**0.59**	**0.58**
4	DOPE:DOPG	50:50	**0.61**	**0.46**
5	DOPC:DOPE:DOPG	24.5:50.5:24	**0.56**	**0.40**
6	DPhPC	1	**0.43**	**0.34**
7	DPhPG:DPhPC	34:66	**0.30**	**0.32**
8	DPhPG:DPhPC	50:50	**0.40**	**0.33**
9	DPhPG:DPhPC	66:34	**0.53**	**0.44**

1.2 Table 2.

Average total yields before and after purification for individual experiments using 100 % DPhPG lipids in PURExpress® systems. The total yield of protein was much smaller in pure DPhPG when compared with other DPh lipid conditions tested. Only repeat 1 (in bold) yielded protein in comparable amounts. Results with DPhPG were thus not presented with other DPh lipid results as only one repeat yielded comparable protein expression yields with other IVTT reactions in different liposome compositions.

**DPhPG**	Repeat	**Total Yield (µg/ 25 µL)**	**Purified Yield (µg/ 25 µL)**
	1	**0.19**	**0.23**
	2	*0.06*	*0.09*
	3	*0.06*	*0.06*
	4	*0.07*	*0.1*

1.3 Table 3.

Average total yields and average purified yields for CL lipid mixes in PURExpress® systems. Total yields are calculated before purification, and purified yields are calculated after sucrose gradient purification, both are presented here as an average of repeated experiments of the same lipid mixes. In the lipid conditions presented here all yields are µg/ 25 µL.

	Lipid Composition	Molar Ratio	**Average Total Yield (µg/ 25 µL)**	**Average Purified yield (µg/ 25 µL)**
1	DOPC:$CL_{E. coli}$	99.5:0.5	**0.55**	**0.50**
2	DOPC:$CL_{E. coli}$	95:5	**0.56**	**0.63**
3	DOPC:DOPE:DOPG:$CL_{E. coli}$	24:51.5:24:0.5	**0.57**	**0.45**
4	DOPC:DOPG:$CL_{18:1}$	50.5:49:0.5	**0.60**	**0.56**
5	DOPC:DOPG: $CL_{18:1}$	53:41.5:5.5	**0.62**	**0.53**
6	DOPE:DOPG:$CL_{18:1}$	72:27.5:0.5	**0.72**	**0.70**

1.4 Table 4.

Average total yields and average purified yields for CL lipid mixes, where SecYEG is present at either 1 in 25, 1 in 50 or 1 in 100 (w/w) protein: lipid ratios. Experiments conducted in Expressway™.

Lipid Composition	Molar Ratio	SecYEG	**Average Total Yield (µg/ 50 µL)**	**Average Purified yield (µg/ 50 µL)**
DOPE:DOPG:$CL_{18:1}$	72:27.5:0.5	1 in 25	**3.8**	**1.2**
DOPE:DOPG:$CL_{18:1}$	72:27.5:0.5	1 in 50	**4.4**	**1.4**
DOPE:DOPG:$CL_{18:1}$	72:27.5:0.5	1 in 100	**4.4**	**1.4**
DOPE:DOPG:$CL_{18:1}$	72:22.5:5.5	1 in 25	**4.0**	**1.6**
DOPE:DOPG:$CL_{18:1}$	72:22.5:5.5	1 in 50	**4.4**	**1.3**
DOPE:DOPG:$CL_{18:1}$	72:22.5:5.5	1 in 100	**4.7**	**1.5**

1.5 Table 5.

All results in EXPRESSway™ with $CL_{\textit{E. coli}}$ lipid mixes, showing individual reactions and repeats where results were omitted. Results that were kept are presented in bold, and results omitted in italics. Results were discarded as highlighted in Methods 2.2.1. Where ≥ 80 % of total protein was lost during purification. In these instances, we considered liposome aggregation to be impacting protein insertion, this was most prevalent with the $CL_{\textit{E. Coli}}$, a natural extract with higher general variability.

**Lipid composition**	**Molar Ratio**	**Total Yield (µg/ 50 µL)**	**Yield Purified (µg/ 50 µL)**
DOPE:DOPC:$CL_{E. coli}$	72:27.5:0.5	*9.3*	*0.51*
		**3.6**	**1.3**
		7.1	*0.27*
		**5.9**	**2.3**
		**5.8**	**2**
		5.5	*0.56*
		**2.5**	**1.5**
		**3.6**	**1.7**
		3.8	*0.14*
		5.3	*1.3*
		4.6	*0.9*
		**4.2**	**3.1**
		**3.8**	**1.5**

1.6 Table 6.

All results in EXPRESSway™ where SecYEG was reconstituted at a 1 in 50 (w/w) protein: lipid ratio, showing individual reactions and repeats where results were omitted. Results that were kept are

presented in bold, and results omitted in italics. Any conditions not presented in this table for SecYEG reconstitutions using various lipids were where no results were required to be omitted.

**Lipid composition**	**Molar Ratio**	**SecYEG**	**Total Yield (µg/ 50 µL)**	**Yield Purified (µg/ 50 µL)**
DOPE:DOPC:CL$_{E. coli}$	72:27.5:0.5	1 in 50	*9.8*	*0.45*
			**3.8**	**1.1**
			7.5	*0.4*
			**6.9**	**2.1**
			**4**	**1.2**
			5.4	*0.3*
			**2**	**0.8**
			**4.1**	**2.2**
			4.8	*0.86*
			11.6	*0.58*
DOPE:DOPC:CL$_{E. coli}$	72:22.5:5.5	1 in 50	**2.5**	**1.0**
			**2**	**0.7**
			7.1	*1.7*
			5.4	*1*

1.7 Table 7.

CL$_{16:0}$ lipid results that fell outside predetermined experimental boundaries, where results omitted are presented in italics. All experiments were conducted using EXPRESSway™ in both conditions where SecYEG was present and absent. In this lipid mix, aggregation of lipids leads low protein recovery after sucrose gradient purification.

**Lipid composition**	**Molar Ratio**	**SecYEG**	**Mean Insertion (%)**	**Total Yield (µg/ 50 µL)**	**Yield Purified (µg/ 50 µL)**	**Protein Recovered after purification (%)**
DOPE:DOPC:CL$_{16:0}$	72:27.5:0.5	no	*0.5*	*7.5*	*0.23*	*3.1*
DOPE:DOPC:CL$_{16:0}$	72:22.5:5.5	no	*2*	*6.7*	*0.56*	*8.4*
DOPE:DOPC:CL$_{16:0}$	72:27.5:0.5	1 in 50	*3.4*	*5.3*	*0.6*	*11.1*
			**20.5**	**7.3**	**1.7**	**23.3**
			0.34	*10.4*	*0.1*	*1.0*
DOPE:DOPC:CL$_{16:0}$	72:22.5:5.5	1 in 50	**18.4**	**5.4**	**1.3**	**24.1**

1.8 Table 8.

SecA dependent ATPase activity of SecYEG when reconstituted into liposomes comprising various lipid mixes (1-8). Activity is quantified as moles of ATP hydrolyzed, per moles of SecA, per minute. Each lipid condition was investigated with ≤ 3 repeats, except 72:22.5:5.5 mol ratios of DOPE:DOPC:CL*E. coli* and 30:70 DOPG:DOPE where n = 2. A graphical representation of this data is shown in Supplementary Figure 3.

	Lipid Composition	Molar Ratio	SecYEG	Moles ATP Hydrolysed, Moles $SecA^{-1}.min^{-1}$
1	E. coli polar lipids	1	**no**	**1.8**
1	E. coli polar lipids	1	**yes**	**3.3**
2	DOPE:DOPG:$CL_{E. coli}$	72:27.5:0.5	**no**	**2.8**
2	DOPE:DOPG:$CL_{E. coli}$	72:27.5:0.5	**yes**	**4.8**
3	DOPE:DOPG:$CL_{E. coli}$	72:22.5:5.5	**no**	**0.6**
3	DOPE:DOPG:$CL_{E. coli}$	72:22.5:5.5	**yes**	**1.5**
4	DOPE:DOPG:$CL_{E. coli}$	70:20:10	**no**	**1.3**
4	DOPE:DOPG:$CL_{E. coli}$	70:20:10	**yes**	**1.8**
5	DOPE:DOPG:$CL_{16:0}$	72:27.5:0.5	**no**	**0.8**
5	DOPE:DOPG:$CL_{16:0}$	72:27.5:0.5	**yes**	**2.1**
6	DOPE:DOPG:$CL_{16:0}$	70:20:10	**no**	**0.5**
6	DOPE:DOPG:$CL_{16:0}$	70:20:10	**yes**	**0.93**
7	DOPC:DOPE:DOPG:$CL_{E. coli}$	24:51.5:24:0.5	**no**	**2.8**
7	DOPC:DOPE:DOPG:$CL_{E. coli}$	24:51.5:24:0.5	**yes**	**3.2**
8	DOPG:DOPE	30:70	**no**	**0.5**
8	DOPG:DOPE	30:70	**yes**	**0.6**

1.9 Table 9.

Percentage insertion efficiency averages for CL*E. coli* lipid conditions where erroneous individual repeats were omitted from complete data sets. Comparison of average insertion efficiencies for where all data included in calculations with averages where data was omitted to reject high aggregation. In each instance the percentage insertion efficiency is presented for both corrected data sets and for data sets where no data has been rejected above a predetermined threshold.

Lipid Composition	SecYEG	Molar Ratio	**Insertion Efficiency (%) without correction**	**Insertion Efficiency (%) with correction**
DOPE:DOPC:$CL_{E. coli}$		72:27.5:0.5	12.3	20.3
DOPE:DOPC:$CL_{E. coli}$	1 in 50	72:27.5:0.5	10.5	16.3
DOPE:DOPC:$CL_{E. coli}$	1 in 50	72:22.5:5.5	17.2	21.1

2 Supplementary Figures

2.1 Figure 1.

Radiolabeled gel corresponding to IVTT with IMVs. Methionine, L-[^{35}S] added to cell-free Expressway™ reaction for incorporation into LeuT during IVTT. Reactions with IMVs were performed in the presence and absence of LeuT template DNA. After purification, both top and bottom fractions were visualized using SDS-PAGE. In conditions where LeuT DNA added, two bands corresponding to LeuT monomer and LeuT dimer were visualized on the gel in both the top and bottom fractions. Bands corresponding to IMVs were present in all conditions (+/- DNA), in both the top and bottom fractions, likely as a result on non-specific incorporation of Methionine, L-[^{35}S] with IMV components.

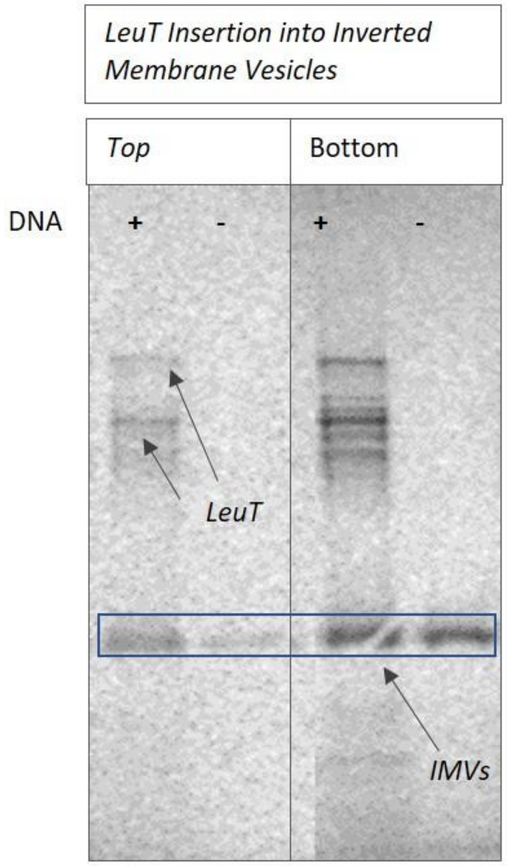

2.2 Figure 2.

Coomassie Brilliant Blue was used to stain an SDS-PAGE gel for SecYEG after protein purification. Enabling visualization of the three distinct subunits; SecY, SecE, SecG highlighted here. SecY = 1332 bp, SecE = 411 bp, SecG = 333 bp. The gel was run using MES running buffer to maximize separation of the subunits.

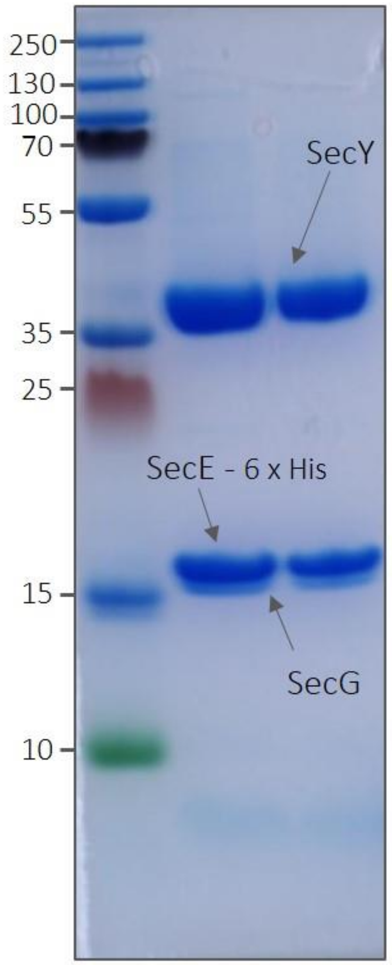

2.3 Figure 3.

SecA dependent ATPase activity of SecYEG when reconstituted into liposomes comprising various lipid mixes (1-8) as seen in Supplementary Table 8. Activity is quantified as moles of ATP hydrolyzed, per moles of SecA, per minute. Numerical data presented in this figure can be found in Supplementary Table 8.

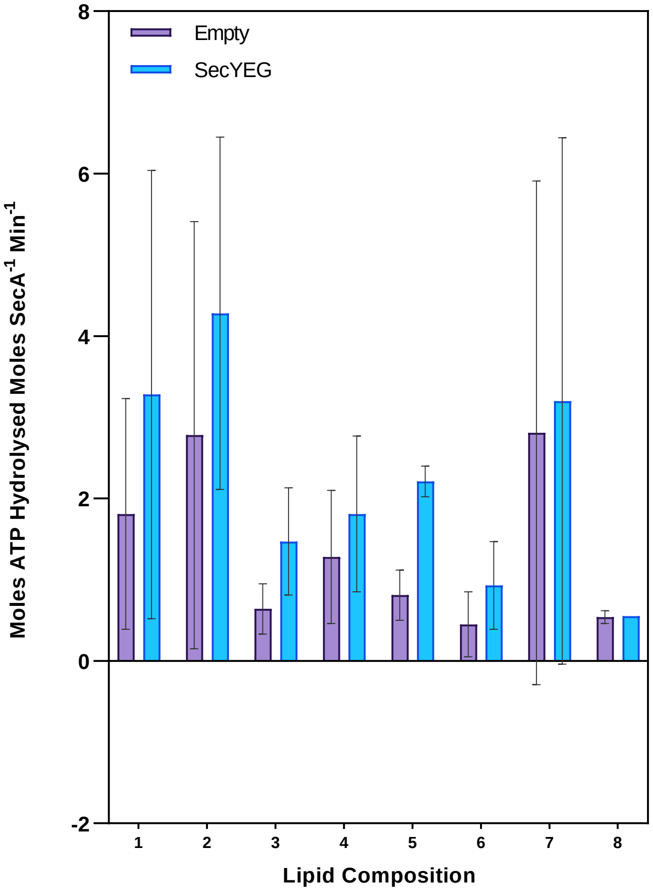

2.4 Figure 4.

Protein recovered between total protein yields and yields after gradient purification, presented as a percentage, is plotted against initial total protein yields after IVTT synthesis. Each EXPRESSway™ repeat result for LeuT synthesis in liposomes containing either $CL_{E.\ coli}$, $CL_{18:1}$ and $CL_{16:0}$ at all concentrations is represented to show the negative correlation between a high initial total yield and low protein recovery after purification. The threshold is illustrated at 20 % to show the cut-off for individual repeats for inclusion in insertion efficiency averages.

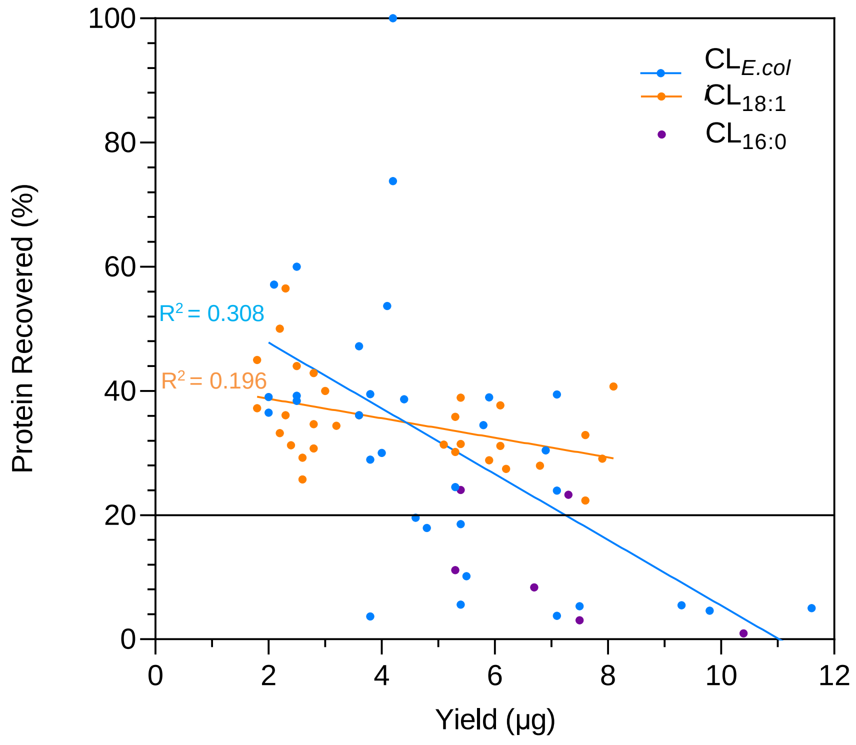

2.5 Figure 5.

A) Insertion efficiency of LeuT in DOPE:DOPG:$CL_{18:1}$ (molar ratio 72:27.5:0.5) liposomes in PURExpress® and EXPRESSway™ IVTT systems, where average percentage insertion was 17.4 ± 3.7 % and 18.7 ± 2.7 % in the two kits respectively. B) Average total initial yields and average yields after gradient purification for LeuT in both PURExpress® and EXPRESSway™ IVTT kits. PURExpress® yields have been doubled to account for the discrepancy in volume differences between the two systems. The average yield for LeuT in PURExpress is 0.72 ± 0.02 $\mu g.25^{-1}$ total yield and 0.70 ± 0.06 $\mu g.25^{-1}$ after gradient purification, this equates to 1.44 $\mu g.50^{-1}$ and 1.4 $\mu g.50^{-1}$ when adjusted for volume size respectively. The average yield for LeuT in EXPRESSway™ is 4.4 ± 0.90 $\mu g.50^{-1}$ and 1.4 ± 0.16 $\mu g.50^{-1}$ for total initial yield and the yield in the gradient respectively.

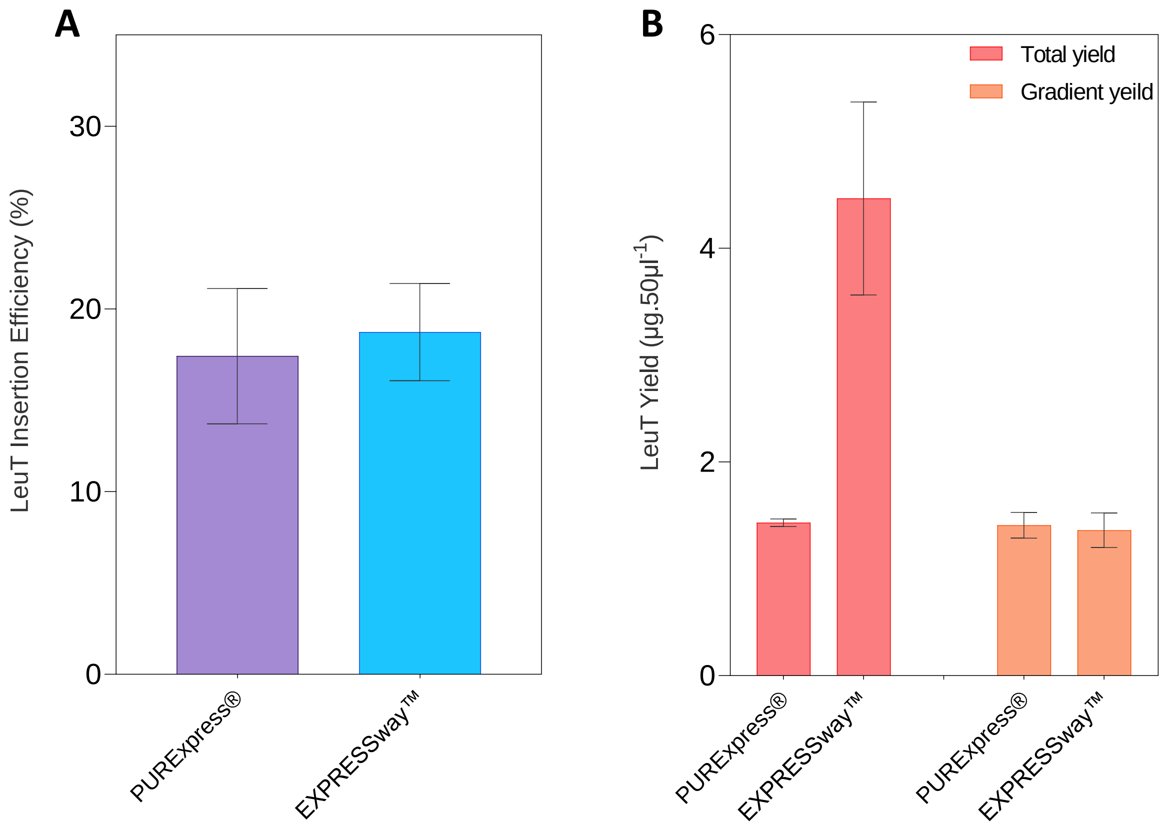

Supplement: Supplementary file 1 [file DataSheet1.pdf]
